# A New Neural Pathway from the Ventral Striatum to the Nucleus Basalis of Meynert with Functional Implication to Learning and Memory

**DOI:** 10.1007/s12035-019-1588-0

**Published:** 2019-04-18

**Authors:** Si Yun Shu, Gang Jiang, Zhaocong Zheng, Lin Ma, Bin Wang, Qiyi Zeng, Hong Li, Shen Tan, Bin Liu, Wood Yee Chan, Sheng Wu, Chunhua Zhu, Changke Li, Peng Wang, Jang-Yen Wu

**Affiliations:** 1 Pediatric Center, Zhujiang Hospital of the Southern Medical University, A- 3103, Building 39, No. 253 Gong-ye Road, Haizhu District, Guangzhou, 510280 Guangdong China; 2 Department of Ear, Nose and Throat, Zhujiang Hospital of the Southern Medical University, Guangzhou, 510282 Guangdong China; 3 Department of Neurosurgery, Fuzhou Central Hospital of Nanjing Military Region, Fuzhou, 350025 Fujian China; 4 Department of Radiology, General Hospital of People’s Liberation Army, Beijing, 100853 China; 5 Department of Neurology, Zhujiang Hospital of Southern Medical University, Guangzhou, 510282 Guangdong China; 6 Emergency Department, Zhujiang Hospital of Southern Medical University, Guangzhou, 510282 Guangdong China; 7 School of Biomedical Sciences, Faculty of Medicine, Chinese University of Hong Kong, Shatin, Hong Kong; 8 Nanjing Junqu Hangzhou Sanatorium, 5 Long-jin Road, Hangzhou, 310007 China; 9 Anesthesia Department of Yue-Bei People Hospital, Shaoguan City, 512026 Guangdong China; 10 The Fifth Affiliated Hospital of Southern Medical University, Guangzhou, 510900 China; 11 Department of Biomedical Science, Charles E. Schmidt College of Medicine, Florida Atlantic University, 777 Glades Road, Boca Raton, FL 33431 USA

**Keywords:** The striatum, The nucleus basalis of Meynert (NBM), Learning and memory, Tract tracing, Y-maze, Immunocytochemistry, Electronic microscopy, Alzheimer’s disease

## Abstract

The cholinergic neurons in the nucleus basalis of Meynert (NBM) are among the first group of neurons known to become degenerated in Alzheimer’s disease, and thus the NBM is proposed to be involved in learning and memory. The marginal division (MrD) of the striatum is a newly discovered subdivision at the ventromedial border of the mammalian striatum and is considered to be one part of the ventral striatum involved in learning and memory. The present study provided evidence to support the hypothesis that the MrD and the NBM were structurally connected at cellular and subcellular levels with functional implications in learning and memory. First, when wheat germ agglutinin-conjugated horseradish peroxidase (WGA-HRP) was stereotaxically injected into the NBM, fusiform neurons in the MrD were retrogradely labeled with WGA-HRP gray-blue particles and some of them were double stained in brown color by AchE staining method. Thus, cholinergic neurons of the MrD were shown to project to the neurons in the NBM. Second, in anterograde tract-tracing experiments where WGA-HRP was injected to the MrD, the labeled WGA-HRP was found to be anterogradely transported in axons from the MrD to the synaptic terminals with dendrites, axons, and perikaryons of the cholinergic neurons in the NBM when observed under an electronic microscope, indicating reciprocal structural connections between the MrD and the NBM. Third, when bilateral lesions of the MrD were injured with kainic acid in rats, degenerative terminals were observed in synapses of the NBM by an electronic microscope and severe learning and memory deficiency was found in these rats by the Y-maze behavioral test. Our results suggest reciprocal cholinergic connections between the MrD of the ventral striatum and the NBM, and implicate a role of the MrD-NBM pathway in learning and memory. The efferent fibers of cholinergic neurons in the NBM mainly project to the cortex, and severe reduction of the cholinergic innervation in the cortex is the common feature of Alzheimer’s patients. The newly discovered cholinergic neural pathway between the MrD of the ventral striatum and the NBM is supposed involved in the memory circuitries of the brain and probably might play a role in the pathogenesis of the Alzheimer’s disease.

## Introduction

The subcortical striatum is a heterogeneous structure including the ventral striatum (nucleus accumbens), the dorsolateral striatum, and the dorsomedial striatum, and is believed to be involved in modulation of complex motional [1] and learning-memory activities [2]**.** The ventral striatum (nucleus accumbens) is known to play a predominant role in association with reward and motivation [3], whereas the dorsolateral striatum is able to formulate stimulus-response strategies such as habit formation [4], body-centered memory including egocentric coordination [5], and unconscious procedural memories that are strengthened during trial-and-error learning [6]. The dorsomedial striatum, similar to the caudate nucleus in humans, is associated with goal-directed learning [7]. Neuropathological evidence indicates decreases of cholinergic interneurons with reduced choline acetyltransferase (ChAT) activities in the striatum of patients with Alzheimer’s disease [8]. Some recent studies have provided behavioral and anatomical evidence to support the notion that associations exist between different brain regions including the striatum and the hippocampus [9–11], the striatum and the prefrontal cortex [12], the striatum and the thalamus [13], the striatum and the amygdala [14], the hippocampus and the prefrontal cortex [15], the hippocampus and the thalamus [16], the prefrontal cortex and the thalamus [17], the hippocampus and the amygdala [18], the nucleus basalis of Meynert (NBM) and the medial temporal lobe system [19], and the amygdala and the thalamus [20], but observations on the direct anatomical connection between the striatum and the NBM are still absent [21, 22].

**Fig. 1:**
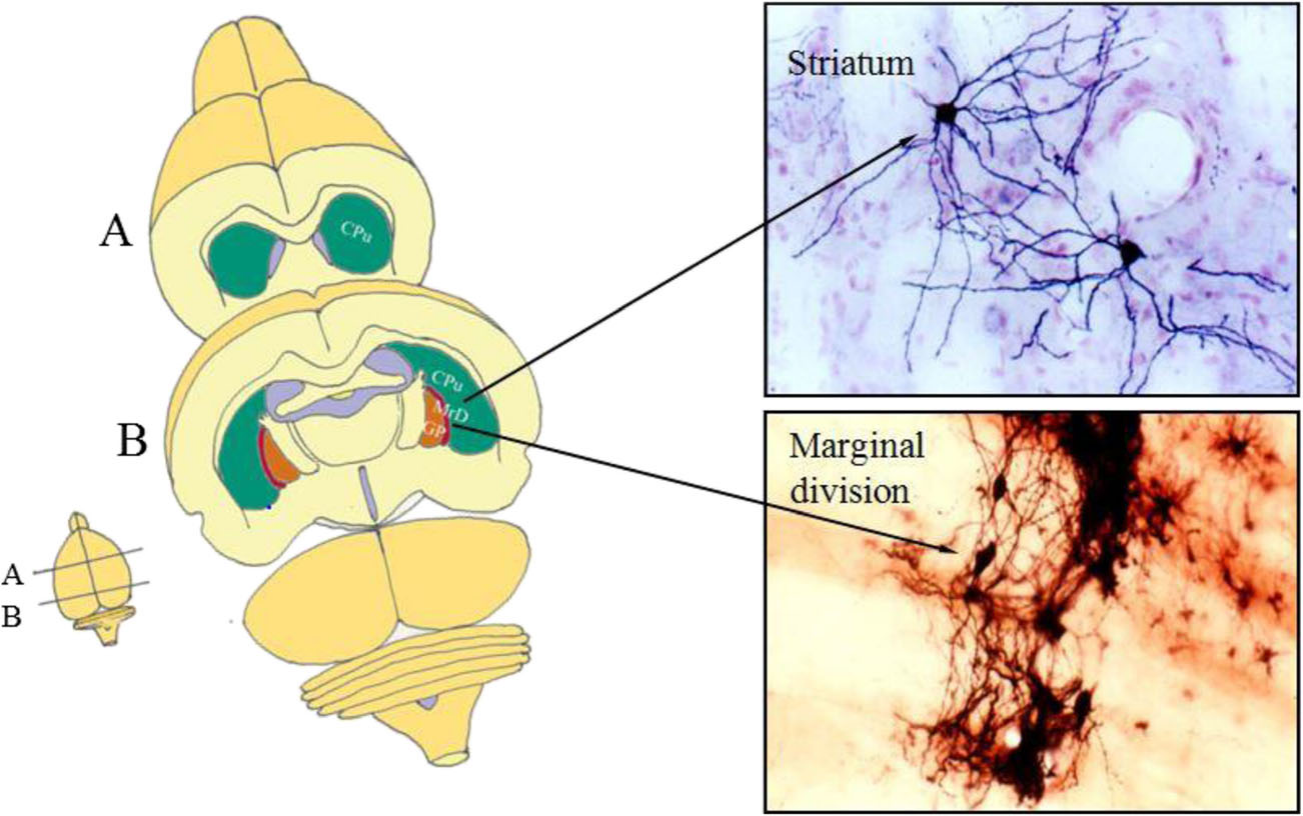
Location and cytoarchitectural characteristics of the MrD in the ventral portion of the striatum in the rat brain. The diagram on the left side indicates two frontal sections (A and B) of the rat brain. The upper right panel shows that the PHA-L-labeled neuronal bodies in the dorsal part of the striatum (caudoputamen, CPu) are mostly of round or triangular shape. The lower right panel illustrates the MrD located in the ventromedial portion of the striatum (CPu) and dorsolateral to the globus pallidus (GP). The MrD consists of a band of fusiform neurons labeled by PHA-L, which distinguishes the MrD from the rest of the striatum (CPu) and the GP. Cpu, caudoputamen of the striatum corpus; GP, globus pallidus; MrD, marginal division of the striatum

Previous studies identified a new brain area, the marginal division (MrD), at the ventromedial border of the striatum in the rat, cat, monkey, and human [23–25]. The MrD is distinguished from the rest of the striatum by its spindle-shaped neurons, specific connections, and dense immunoreactivities of neuropeptides and monoamines in fibers, terminals, and neuronal somata [23–26] (Fig. 1). The 5′-nucleotidase activity is densely expressed in the developing rodent MrD [27], whereas the a2-adrenergic receptors are expressed more strongly in the MrD than in the rest of the rat striatum [28]. Furthermore, it has been shown that the pedunculopontine nucleus sends to massive afferents to the MrD of the squirrel monkey [29], and that the MrD is connected to the interstitial nucleus of the posterior limb of the anterior commissure [30]. The MrD has been suggested to be one of the five components of the ventral striatum [31]. Behavioral tests and physiological experiments showed that the MrD contributes to learning and memory or pain reception in the rat [32, 33]. Reduction in the capacity of learning and memory was observed in the Y-maze test after chemically induced bilateral lesions of the MrD of the rat [32]. The c-Fos protein was expressed in the hippocampus, dentate gyrus, amygdala, and the forebrain cortex after injection of kainic acid into the MrD, demonstrating the functional connections between the MrD and these structures [34]. In addition, the MrD was found to have fiber connections with the amygdaloid nucleus [35] and the bed nucleus of the striatum terminals [36]. Moreover, it was also found that the efferent fibers from the MrD projected to the most caudal part of the globus pallidus, where the NBM was located. In the human brain, the association of the MrD with learning and memory has been demonstrated with both pathological case reports and functional MRI analyses [25], thus leading to the suggestion that the MrD of the neostriatum is a subcortical memory center and a new component of the limbic system [37]. Substance P which was expressed in the MrD was found to play a role in learning and memory and mediated through the neurokinin 1 receptor in rats [38]. Interactions between the hippocampus and the striatum were reported during episodic encoding [39]. Immunohistochemical study found that the distribution of muopioid receptor in the MrD was different from those in other parts of the neostriatum [40]. Different expression patterns of microRNAs were also observed in the MrD and the hippocampus of the rat [41]. Previous studies suggested that the MrD and the hippocampus may play different roles in learning and memory through different neurotransmission mechanism [42]. New functional and structural pathways related to learning and memory through the hippocampus, the amygdala, and the MrD in rats have been demonstrated [43].

Unlike the aforementioned relations between the striatum and various brain structures, less attention has been paid to the connection between the striatum and the NBM. The NBM as the major source of cholinergic innervation of the cerebral neocortex belongs to the basal forebrain cholinergic system [44]. As the degeneration of the cholinergic projections from the basal forebrain to the neocortex and the hippocampus has been found to be correlated with memory decline in Alzheimer’s disease [45], it is proposed that the striatum may also be associated with the NBM during the process of learning and memory [46]. Although cognitive deficits are usually considered to be attributed by the loss or degeneration of cholinergic neurons in the NBM [46, 47], some reports have cast doubt on this proposition [48, 49]. Hence, both associations between the striatum and the NBM and the cognitive deficits and loss of synapses in the NBM need to be further examined.

In the present study, we carefully dissected detailed anatomical connections between the MrD and the NBM with wheat germ agglutinin-conjugated horseradish peroxidase (WGA-HRP) tract-tracing, histochemical staining, electronic microscopy, and immunoelectronic microscopic techniques. The mechanism underlying the learning-memory process associated with the connections between the MrD and the NBM was also demonstrated through lesions of the MrD followed by studies in the Y-maze behavioral test with tract-tracing, electronic microscopy, and immunoelectronic microscopic techniques.

## Materials and Methods

### Animals

A total of 100 male Sprague-Dawley rats weighing 200–250 g were obtained from Beijing Research Center for Experiment Animals, Beijing, China, and used in this study. The rats were housed individually at a constant temperature of 25 °C with ad libitum access to food and water in a 12:12-h light/dark cycle. They were randomly divided into two large groups: behavioral test group (*n* = 80) and non-behavioral test group (*n* = 40). Rats in the non-behavioral test group were randomly divided into four subgroups: (1) control group with no treatment (*n* = 5); (2) WGA-HRP-treated group with 1% WGA-HRP injected into the MrD or NBM (*n* = 15, *n* = 15); (3) kainic acid (KA)-treated group with 0.1% KA injected directly into the MrD (*n* = 10); and (4) normal saline (NS)-treated group with NS injected into the MrD (*n* = 10). For the behavioral test group, after the pre-test in the Y-maze (see below), rats (*n* = 53) were randomly divided into five subgroups: (1) Bilaterally lesioned MrD group (*n* = 18); (2) unilaterally lesioned MrD group (*n* = 10); (3) bilaterally lesioned caudoputamen (CPu) group (*n* = 10); (4) bilaterally lesioned NBM group (*n* = 10) and (5) bilaterally saline-injected MrD group (*n* = 5). All experimental procedures on animals were approved by the Institute for Animal Care of the Southern Medical University. All efforts were made to minimize the number of animals used and any pain they might experience during the course of the investigation.

### Histochemical and Tract-Tracing Methods

One percent WGA-HRP (Sigma) (0.2 μl) was stereotaxically injected into the NBM or the MrD of the rat’s brain through a micropipette as the retrograde and anterograde tracers in the non-behavioral test group. Two days after WGA-HRP injections, the rats in the non-behavorial test group were anesthetized with 10% chloral hydrate (3.5 ml/kg, intraperitoneal (i.p.)). Under deep anesthesia, the rats were perfused through the aorta with 200 ml 0.9% saline followed by 200 ml fixative solution containing 3% paraformaldehyde and 0.25% glutaraldehyde in 0.02 M sodium phosphate buffer (pH 7.4, 4 °C) within 1.5 h. After fixation by perfusion, the brains were removed and immersed in the same fixative solution for 4 h before they were transferred to and immersed in a phosphate-buffered 20% sucrose (0.02 M sodium phosphate buffer, pH 7.4, 4 °C) until the brains sank to the bottom of the solution. Brains were sectioned coronally on a vibratome (LKB, Sweden) at thickness of 50 μm. The sections were immersed in 0.01 M phosphate buffer (pH 7.4, 4 °C) for Nissl staining and acetylcholine esterase (AchE) staining or AchE-TMB (3,3′, 5, 5′-tetramethylbenzidine) double staining. The WGA-HRP was visualized after reacting with 3,3′,5, 5′-tetramethylbenzidine (TMB) (Sigma) according to the method described by Mesulam [50]. The sections were prepared and examined with a light microscope (Olympus).

Nissl staining was carried out with 0.1% cresyl violet for examination of the Nissl bodies and morphology of neuronal bodies in the brain. Cryostat sections were mounted on slides and stained with 0.1% cresyl violet, then dehydrated, cleared, and coverslipped. AchE staining was carried out as follows [51]: after five changes of acetate buffer (1 min each), sections were treated with 1% ammonium sulfide solution for 1 min followed by five changes of 0.1 M sodium nitrate (1 min each). Sections were then exposed to 0.1% silver nitrate for 1 min followed by five changes of 0.1 M sodium nitrate (1 min each). Free-floating sections were rinsed in the acetate buffer, mounted from acetate buffer onto subbed slides, air-dried, dehydrated, cleared, and coverslipped. Sections were counterstained before dehydration. AchE-TMB double staining was carried out as follows [52]: after incubation in AchE staining solution for 60 min, sections were rinsed in dH_2_O for six times (1 min each). Then they were reacted with 1% ethylene diamine tetraacetic acid (EDTA) solution for 1 min, and further incubated in 1% H_2_O_2_ solution for 20 min, and then glucose saline (GS, 40 mg/100 ml) and glucose oxidase (GOD, 1 mg/100 ml) were added to the incubation solution for an additional 20 min. The color reaction was visualized using 10% potassium ferricyanide after the sections were rinsed in the acetate buffer for six times (30 min each). After color development, the sections were air-dried, dehydrated, cleared, and coverslipped.

### Lesion

All rats were anesthetized with an i.p. injection of 10% chloral hydrate (3.5 ml/kg). After anesthetized, they were subsequently placed on a stereotaxic platform. Their scalps were opened longitudinally with a scalpel and three small holes were drilled in the skull at the coordinates indicated in the bregma system [53]. Glass micropipettes were introduced through the small holes into the brain to perform microinjection of KA, WGA-HRP, or saline (0.2 μl each). The coordinates were 1.4 mm posterior to the bregma, 4.2, 5.0, or 3.2 mm lateral to the midline, and 5.3, 5.0, or 6.6 mm ventral from the skull surface for the MrD, CPu, or NBM injection, respectively. After each injection, the needle was left in place for 10 min before slowly retracted.

### Behavioral Test

An electric Y-maze was chosen. This Y-maze uses light as the conditional visual stimulus combined with the avoidance of electric foot-shock pain reinforcement to test associative learning and declarative memory, commonly known as foot shock-motivated brightness discriminating Y-maze test [54]. The Y-maze has three arms with metal wires on their bottom to deliver electric shocks and lights at their ends. When the foot-shock avoidance test began, one arm with the light on (light zone) was the shock-free area, whereas the other two arms with the light off (dark zone) were areas with electric shocks. Electric shocks were delivered to any of these three arms during the test. Rats preferred to enter the dark zones at the beginning of the test. After receiving a foot shock, most of the rats soon learned to escape from the dark to the light zone to avoid electric shocks. During the test, the rats were considered to be able to learn and remember the correct route of escape from the electric shock if they could run to the light zone within 10 s after the light shifted from one to another arm of the Y-maze. The number of correct escapes (running to the light zone within 10 s) in 30 electric shocks was used to quantify memory. All rats (*n* = 80) were tested twice before carrying out the behavioral tests and only those who passed the first test with at least 10 correct escapes and passed the second test with at least 15 correct escapes were used in the subsequent behavioral tests. Two days later after passing the two preliminary tests, the rats in the above groups were tested in the foot-shock avoidance Y-maze with simultaneous changes in the light stimulus. After this avoidance test, the rats who were able to learn that the light end was safe were chosen (*n* = 53) and divided randomly into five subgroups as indicated in the “Animals” section. The rats were then stereotaxically injected with 0.1% KA (0.2 μl) into the MrD bilaterally or unilaterally, the CPu bilaterally, or the NBM bilaterally to produce the specific lesions. Saline was injected bilaterally into the MrD of the control animals. The avoidance tests were carried out again with the Y-maze 2 days after the lesion.

### Electron Microscopic Examinations

Small pieces of the NBM were cut from the AchE-histochemically stained brain sections and WGA-HRP-stained brain sections under a microscope. After postfixed in 2.5% glutaraldehyde for 2 h, the tissues were washed in phosphate buffer and transferred into 0.5% osmium tetroxide at 4 °C for 1 h, and then rinsed in distilled water and dehydrated through a graded series of ethanol. The tissues were incubated in 1% uranyl acetate dissolved in 70% ethanol, processed with propylene oxide, embedded, and then mounted. Ultrathin sections were cut on a LKB-Nova Ultratome (Sweden), stained with 1% lead citrate, and examined under a JEM-1200 (Japan) transmission electron microscope.

### Data Analysis

Statistical analyses were performed with SPSS 17.0 software by SPSS, Inc. All data were presented as means ± SD. Multiple samples were compared using analysis of variance (ANOVA). Multiple means were assessed statistically with paired-samples *t* test. Significant differences were set at *p* < 0.05.

## Results

### Efferent Projections from the MrD to the NBM Shown by WGA-HRP Retrograde Tracing Method

**Fig. 2:**
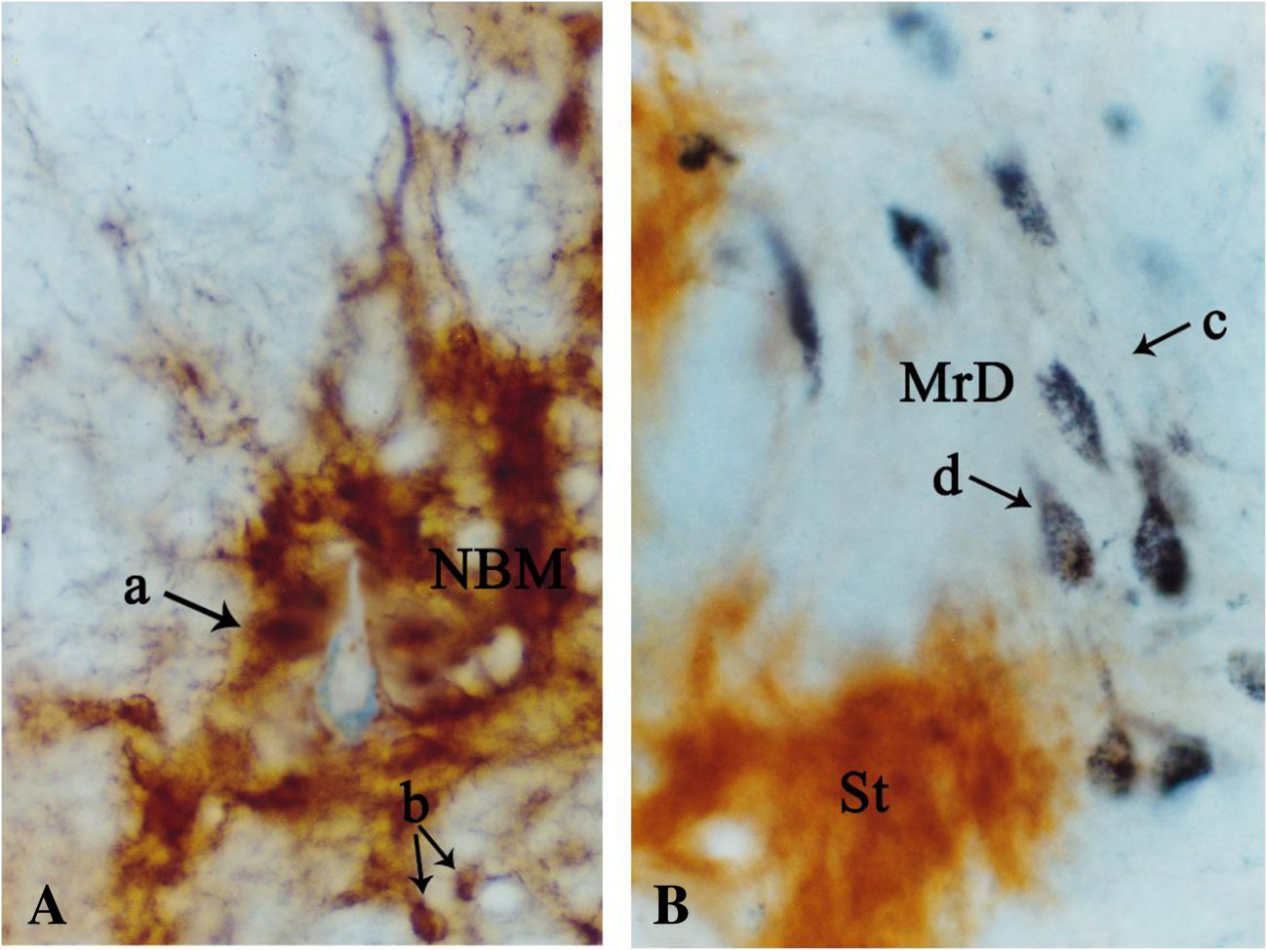
Determination of the projection from the MrD to the NBM by WGA-HRP retrograde tracing method. **a** The WGA-HRP injecting site in the NBM is marked (arrow a) where little WGA-HRP tracer shown as blue substance could be detected. Two cholinergic neurons of NBM were observed at the right lower corner (arrow b). **b** WGA-HRP stained as gray-blue particles retrogradely labeled fusiform somata of neurons in the MrD of the ventral striatum (arrow c) in the rat brain. Some of the WGA-HRP-labeled neurons were stained with brown color background, which were AchE-WGA-HRP double-labeled neurons (arrow d). The AchE-WGA-HRP double-labeled neurons were cholinergic neurons in MrD, which projected to the NBM. MrD, marginal division of the striatum; NBM, basal nucleus of Meynert. Magnification = ×400

**Fig. 3:**
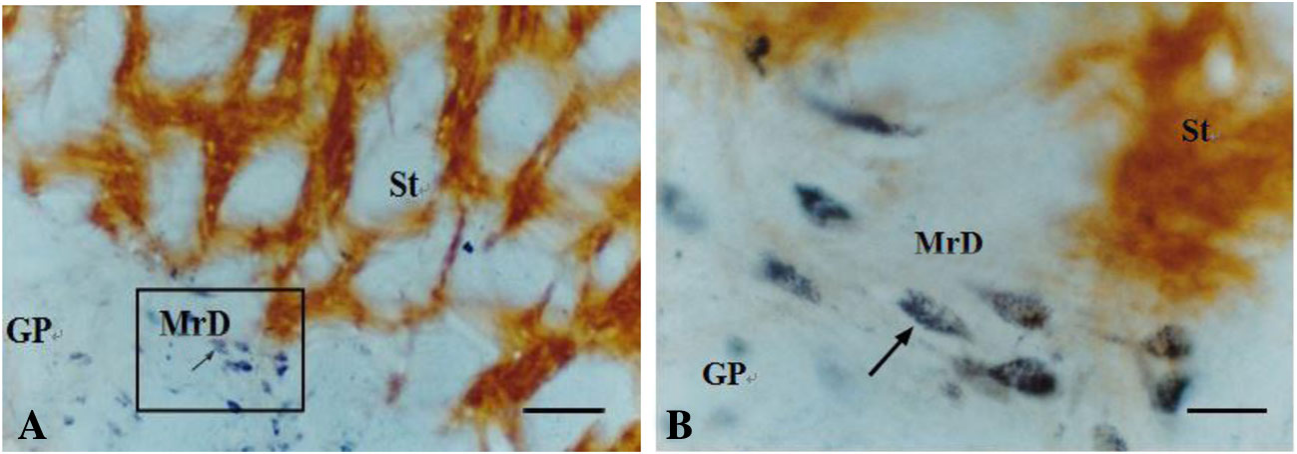
The projection from the MrD to the NBM by WGA-HRP retrograde tracing method. The conditions were the same as in Fig. 2**. a** WGA-HRP stained as gray-blue particles retrogradely labeled fusiform somata of neurons (arrows) in the MrD of the ventral striatum in the rat brain. **b** High magnification of the boxed area in **a**. Some fusiform neurons were double-stained in brown color with some blue particles in their cell bodies. This AchE-WGA-HRP double-stained neurons were cholinergic neurons in the MrD which project to NBM. GP, globus pallidus; MrD, marginal division of the striatum; St, striatum; scale bar = 400 μm (**a**) and 100 μm (**b**)

**Fig. 4:**
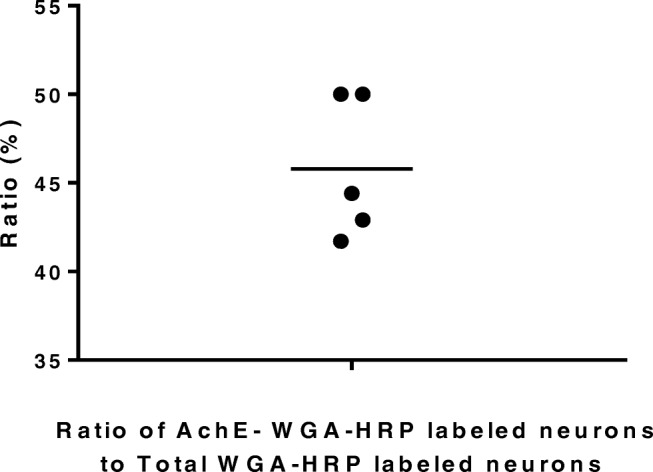
A dot plot showing the ratio as percentage of AchE-WGA-HRP double-labeled neurons to Total WGA-HRP-labeled neurons

WGA-HRP technique is a valuable tract-tracing reagent for studying the nervous connections in the central nervous system. The WGA-HRP could be transported within nerve fibers both anteriorly and retrogradely. TMB staining is a very sensitive method to show the transported WGA-HRP. The WGA-HRP was stained in gray-blue color particles within the plasma of the neuronal cell body or in the nervous fiber terminals by TMB staining. We used the WGA-HRP to trace the connection between the MrD and NBM. Following the stereotaxic injection of WGA-HRP into the NBM, little WGA-HRP detected as the gray-blue substance was observed at or near the injection site (Fig. 2a), whereas the retrogradely transported WGA-HRP which appeared as gray-blue particles was found prominently in some fusiform neurons in the MrD (Fig. 2b) suggesting that WGA-HRP present at the MrD site is not due to non-specific uptake of the injected WGA-HRP tracer but due to a direct projection pathway between NBM and MrD. The retrogradely transported WGA-HRP-labeled fusiform neurons were observed in all five rats. There are eight retrogradely transported WGA-HRP-labeled neurons in the MrD of the striatum in the boxed area in one rat as shown in Fig. 3. The retrogradely transported WGA-HRP-labeled neurons in brain sections of the other four rats were 12, 9, 7, or 10, respectively. Thus, the mean ± SD of WGA-HRP-labeled neurons is 9.2 ± 1.92. Some of WGA-HRP-labeled neurons are also labeled with AchE. The ratio of AchE-WGA-HRP double-labeled neurons to Total WGA-HRP-labeled neurons was 0.500, 0.417, 0.444, 0.429, and 0.500 with the mean ± SD as mean 0.458 ±  0.040 (Table 1). A dot plot showing the ratio as percentage of AchE-WGA-HRP double-labeled neurons to Total WGA-HRP-labeled neurons is shown in Fig. 4. The result showed definitely the presence of efferent projections from the MrD to the NBM. The brown substrate was stained by AchE method to demonstrate the cholinergic containing neurons and nerve fibers. The results demonstrated that the MrD contains cholinergic neurons and some of these cholinergic neurons projected to NBM.

**Table 1:** Ratio of AchE-WGA-HRP double-labeled neurons to Total WGA-HRP-labeled neurons in the MrD of the striatum. Ratio of AchE-WGA-HRP double-labeled neurons to Total WGA-HRP-labeled neurons in percent

Total WGA-HRP-labeled neurons in the box Number of animals: 5	AchE-WGA-HRP double-labeled neurons	Ratio of AchE-WGA-HRP double-labeled neurons to Total WGA-HRP-labeled neurons (%)
Animal 1—8	4	50.0
Animal 2—12	5	41.7
Animal 3—9	4	44.4
Animal 4—7	3	42.9
Animal 5—10	5	50.0

The mean ± SD as mean 0.458 ± 0.040

### Loss of Fusiform Neurons in the MrD After KA Injection Shown by Nissl Staining

**Fig. 5:**
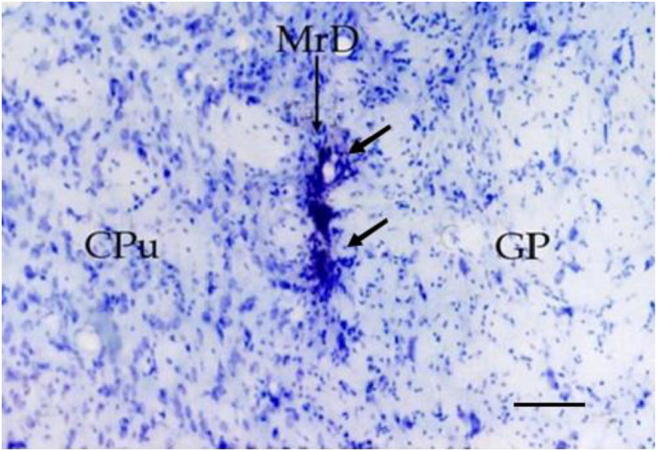
Loss of fusiform neurons in the MrD after KA injection shown by Nissl staining. At the injection site, fusiform neurons in the MrD (upper longer arrow) were lost (pointed by two shorter parallel arrows), while the morphology of neurons in the adjacent striatum (caudoputamen, CPu) and globus pallidus (GP) is still normal. Scale bar = 100 μm

The KA is a toxin to neurons in the brain. We stereotaxically injected 0.1% KA (0.2 μl) into the MrD bilaterally or unilaterally, the CPu bilaterally, or the NBM bilaterally to produce the specific lesions. Only 5 out of 15 KA-injected rats were injected right in bilateral MrD and 8 of 15 rats were injected in NBM precisely. The saline as a control reagent was injected bilaterally into the MrD in the control animals. The fusiform neurons which were parallel in the ventromedial border of the striatum were lost and were replaced with a large number of infiltrated microglia in the sections through the KA injection site in the MrD, while the morphology of neurons in the adjacent striatum and globus pallidus was still normal (Fig. 5). The neurons of the CPu and the NBM were lost as well in the KA-injected group. No obvious histopathological changes were found in the sections of the rats brains in the saline-injected group. The results indicate that the KA injections had injured the neurons in the MrD or CPu or NBM of the rat brains, respectively.

### Identification of AchE-Containing Neurons and Substrate in the MrD, Striatum, and NBM

**Fig. 6:**
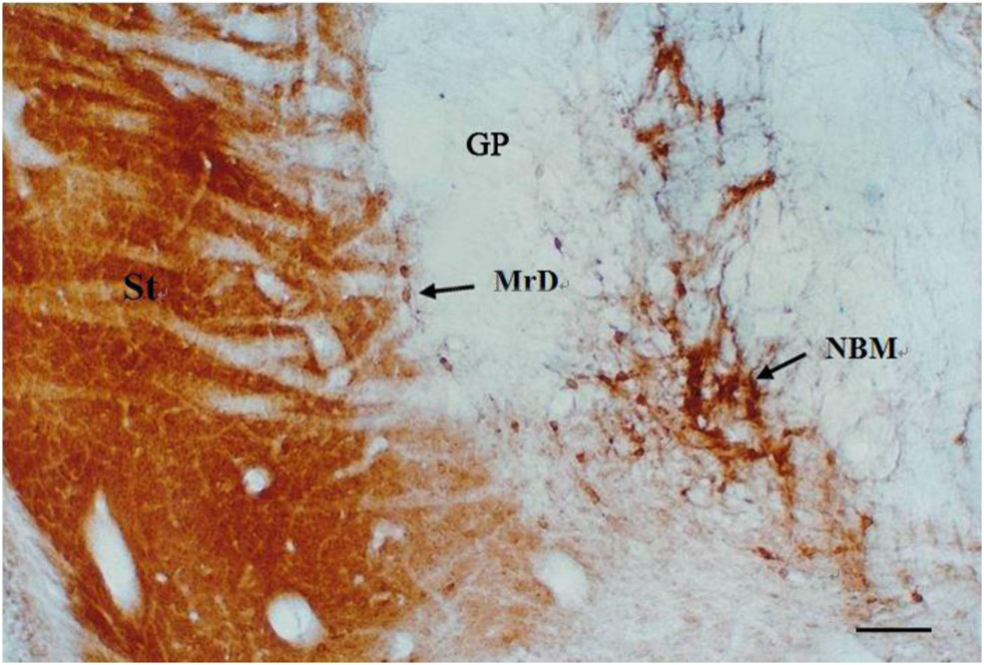
Identification of AchE-containing neurons and neuronal processes in the MrD, striatum, and the NBM. The cholinergic neurons and nerve fibers were stained in dark brown color by AchE staining method. A row of cholinergic medium-size fusiform neurons stained by AchE method in brown color was observed in the MrD of the striatum (black arrow on the left). The large multi-angular cholinergic neurons in the NBM were heavily stained by AchE method in brown color (black arrow on the right). The rest portion of the striatum (St) is fully filled with AchE-positive substrates. MrD, marginal division of the striatum; NBM, nucleus basalis of Meynert; St, striatum; scale bar = 400 μm

The cholinergic neurons in sections of the brain were stained in brown or dark brown color by the AchE method. The dorsal and lateral striatum in the rat brain was heavily stained in brown color fully by AchE staining method of the saline-injected group, and the staining was so heavy that individual neurons were not easily discernible (Fig. 6). Some large multi-angular cholinergic neurons in the NBM were also heavily stained with AchE method. A band of medium-size AchE positives-stained cholinergic fusiform neurons was observed in the MrD with their long axes arranged in parallel to the ventromedial border of the striatum (Fig. 6). The results indicated that both MrD of the striatum and NBM contain cholinergic neurons and nervous fibers.

### Demonstration of Degenerated Terminals in the NBM Identified by the Electron Microscope After KA Injected into the MrD

**Fig. 7:**
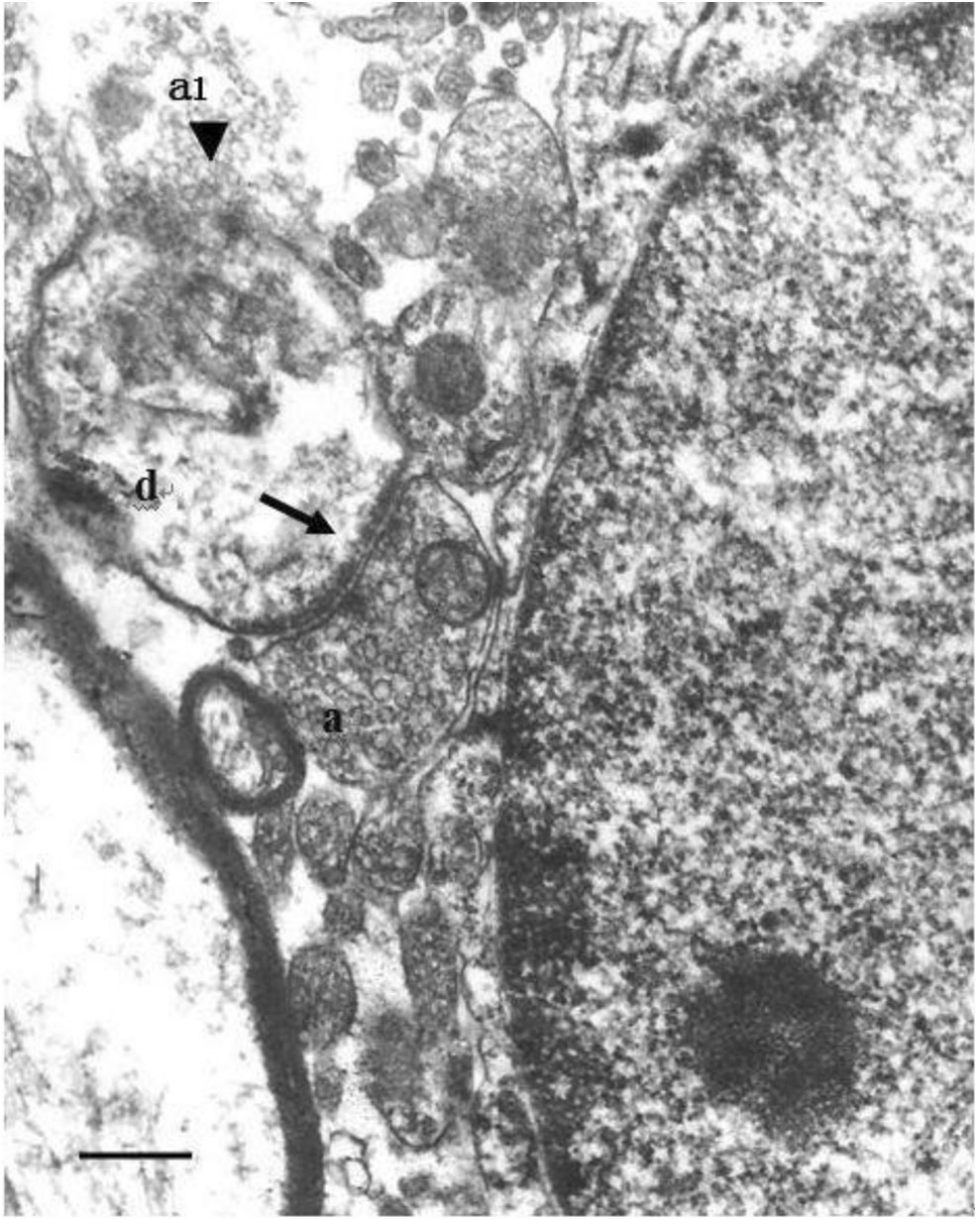
Demonstration of degenerated terminals in the NBM after KA injection into the MrD—electron microscopic analysis. Anterograde degenerative axon terminals are observed in the NBM following KA injection to the MrD of the rat brain. A terminal (a) with synaptic vesicles and swollen mitochondria is found to form an asymmetric axo-dendritic synapse (arrow) on a dendrite (d). A completely damaged axon (a1, black triangle) with decreased numbers of synaptic vesicles and a disrupted axon membrane is found just above the dendrite (d). A cholinergic neuron with a big nucleus in the NBM is occupied the right panel of the figure. MrD, marginal division of the striatum; NBM, nucleus basalis of Meynert; scale bar = 0.5 μm

The small pieces of the NBM were cut from sections of the KA-injured MrD of the rat brain to investigate the degenerated terminal in the synapses of the NBM. These small pieces of tissues were incubated in 1% uranyl acetate dissolved in 70% ethanol, processed with propylene oxide, embedded, and then mounted. The embedded and mounted tissues were cut as ultrathin sections by a LKB-Nova Ultratome (Sweden). The ultrathin sections were examined under a JEM-1200 (Japan) transmission electron microscope for investigating the degenerating terminals projected from the MrD synapsed on the AchE-positive neurons of NBM. Under the transmission electron microscope, iron sulfide products with high electron densities from the AchE staining reaction were found scattered in the neurons, suggesting that these neurons were cholinergic neurons. Some axon terminals which showed substances with high electron densities and round synaptic vesicles were observed in the synapses of AchE neurons in the NBM. The degenerated terminals from the MrD which exhibited decreased numbers of synaptic vesicles, increased electron densities, and mitochondrial deformation or aggregation were observed in the synapses of the NBM (Fig. 7). These terminals formed the axo-somatic, axo-dendritic, axo-axonic, or complex synapses with cholinergic neuronal bodies, dendrites, and axons. Since these degenerated terminals were observed in the NBM after injection of KA to the MrD, this observation suggested that the degenerated terminals were projected from the KA damaged MrD to the NBM.

### Demonstration of WGA-HRP Anterogradely Labeled Neural Terminals from MrD to NBM by Electron Microscopy

**Fig. 8:**
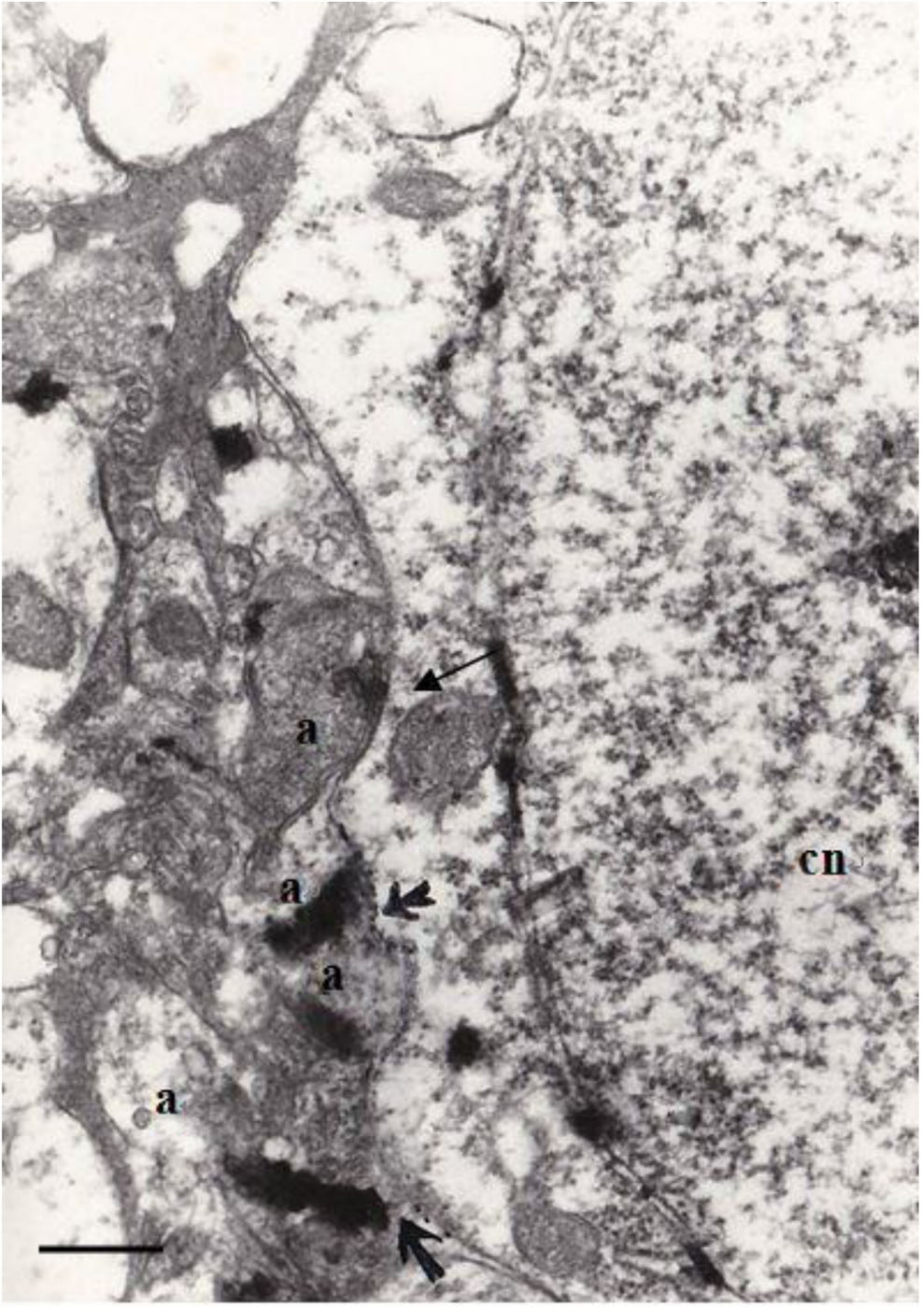
Electron photomicrography showing anterograde WGA-HRP-labeled terminals projected from the MrD to the NBM after WGA-HRP injection in to the MrD. Following WGA-HRP injection to the MrD, anterogradely transported WGA-HRP, which appeared as rod-like electron dense substances, labeled axon terminals (a) from the MrD to form symmetric axo-somatic synapses (arrows) with a cholinergic neuron (cn) of the NBM. MrD, marginal division of the striatum; NBM, nucleus basalis of Meynert; scale bar = 0.5 μm

The WGA-HRP could be transport within nerve fibers both anteriorly and retrogradely. TMB staining is a very sensitive method to show the transported WGA-HRP. The WGA-HRP was stained in gray-blue color particles within the plasma of the neuronal cell body or in the nervous fiber terminals. The WGA-HRP anterogradely transported in the terminals of NBM from the axons of MrD. The TMB-stained WGA-HRP displayed as dark rod-like crystals in the labeled axonal terminals under the transmission electron microscope, which were observed in synapses of the NBM after WGA-HRP was injected into MrD. These anterogradely labeled neural terminals formed symmetric axo-somatic synapses with cholinergic neurons of the NBM under the electron microscope (Fig. 8).

### Behavioral Tests

We used an electric Y-maze to test the behavioral ability of the rat. This Y-maze uses light as the conditional visual stimulus combined with the avoidance of electric foot-shock pain reinforcement to test associative learning and declarative memory, commonly known as foot shock-motivated brightness discriminating Y-maze test [54]. The light-foot shock avoidance reaction is based on the memory-related conditional reflex, and used to test the memory function of the animal. The Y-maze has three arms with metal wires on their bottom to deliver electric shocks and lights at their ends. When the foot-shock avoidance test began, one arm with the light on (light zone) was the shock-free area, whereas the other two arms with the light off (dark zone) were areas with electric shocks. Electric shocks were delivered to any of these three arms during the test. Rats all preferred to enter the dark zones at the beginning of the test. After receiving a foot shock, most of the rats soon learned to escape from the dark to the light zone to avoid electric shocks. After several shocks, rats learned and remembered that the light zone was a safe area and hence they ran directly to the light zone whenever the light was shifted from one area to the other. During the test, the rats were considered to be able to learn and remember the correct route of escape from the electric shock if they could run to the light zone within 10 s after the light shifted from one to another arm of the Y-maze. The number of correct escapes (running to the light zone within 10 s) in 30 electric shocks was used to quantify memory. The behavioral test was performed with the Y-maze in the preliminary tests and also 2 days after the treatment with KA. The learning and memory scores of the rats were expressed by the total numbers of shocks required for rats to choose correctly 9 out of 10 times. No significant differences in the learning and memory scores among rats of different treatment groups were observed before the treatment (*p* > 0.05). The total number of shocks required for the 90% positive response was increased significantly after bilateral KA lesions of the MrD (*n* = 12) or NBM (*n* = 6) (*p* < 0.05), from 15.58 ± 2.02 to 28.58 ± 2.02 or 15.00 ± 1.41 to 26.83 ± 1.33, respectively (Table 2). No significant differences were found between these two groups (*p* > 0.05). Furthermore, no differences in the number of total shocks required for the 90% positive response before and after lesions in the groups of bilateral KA lesions of the caudoputamen (neostriatum) or unilateral KA lesions of the MrD as observed in the saline-injected control group (*p* > 0.05) (Table 2). These results suggested that bilateral lesions of the MrD or NBM led to the decrease of learning and memory ability.

**Table 2:** Total number of shocks required for attaining a 90% correct rate in the Y-maze test before and after the KA treatment

			Total number of shocks^a^	
Group	*N*	Before treatment	After treatment	*p* value
Bi-MrD lesion	12	15.58 ± 2.02	28.58 ± 2.02	< 0.05
MrD lesion	7	15.08 ± 1.29	16.57 ± 0.98	> 0.05
Bi-CPu lesion	8	15.88 ± 1.25	16.25 ± 1.39	> 0.05
Bi-NBM lesion	6	15.00 ± 1.41	26.83 ± 1.33	< 0.05
Control	4	15.75 ± 1.26	16.00 ± 1.15	> 0.05

^a^Values are expressed as mean **±** SD

## Discussion

The results of the present study demonstrated that the MrD and the NBM were structurally connected at both cellular and subcellular levels using a combination of histochemical localization, tract-tracing, electron microscopy, and immunoelectronic microscopy techniques. WGA-HRP was employed to identify the direct connections between neurons, because it could be transported both anterogradely and retrogradely between the neuronal somata and their terminals. Following WGA-HRP injection to the NBM, positive gray-blue particles of WGA-HRP were retrogradely transported within fusiform neurons in the MrD, demonstrating efferent projections from the MrD to the NBM. Conversely, after WGA-HRP injection to the MrD, WGA-HRP labeling of axon terminals was found in the synapses of cholinergic neurons in the NBM under an electron microscope, lending support to the notion that the MrD and the NBM were reciprocally connected.

The Y-maze test has been widely used for identification of discrimination learning, spatial alternation tasks, and working and reference memory [55, 56]. With this method, spatial memory performances and various exploratory behaviors could be assessed quickly [57]. Our previous studies have also shown that when the MrD was stimulated by KA [34], the c-Fos protein was expressed in various parts of the limbic system [58] including hippocampus, dentate gyrus, amygdala, and the forebrain cortex. These results indicated the functional connection between the MrD and the limbic system. In addition, we have also observed the structural connections between the MrD and some areas of the limbic system [24, 35, 59–61]. In the present study, after KA injection to the MrD, anterograde degenerating axon terminals were found in the synapses of cholinergic neurons in the NBM under the electron microscope. No differences among learning and memory scores in rats of different treatment groups were found before stereotactic injections in the MrD or NBM, but the total number of shocks required for the 90% positive response was increased significantly after bilateral KA lesions of the MrD or NBM, and no significant differences were found between the MrD-lesioned and the NBM-lesioned groups. These results suggested that the bilateral lesions of the MrD led to the decrease of learning and memory ability, which supports the proposition that the structural connection between the MrD and the NBM plays a role in the learning and memory function.

The brain constitutes about 2% of the body weight, requires about 17% of the normal cardiac output, and consumes about 20% of the oxygen utilized by the entire body. The striatum is located in the central position of the brain which is supplied by branches of lenticulostriate arteries arising directly from the proximal portion of the middle cerebral artery [62]. Thus, the ventral striatum derives its blood and oxygen supply more quickly and directly from the heart in comparison to other portions of the brain, and probably carries out important functions of the brain including learning and memory. Based on the findings in the present study and other observations [14, 27, 28, 35], the afferent and efferent signals could be transported from the MrD to all its connected areas. For instance, the efferent striatonigral fibers from the MrD terminated at the caudal border of the substantia nigra, the pars reticulate [60]. The afferent fibers from the amygdaloid nucleus and the bed nucleus of the stria terminated to the MrD [35, 61]. The MrD and the hippocampus have been proposed to have different roles in learning and memory [42], partly based on the finding on the new pathways among the hippocampus, the amygdala, and the ventromedial region of the striatum in rats [43]. The results obtained from the present study with tract tracing and immunoelectronic microscopy showed a new pathway where neurons of the MrD projected to the NBM and their axon terminals had synaptic connections with the cholinergic neurons of the NBM. Lesion of the new pathway from the MrD to the NBM in rats resulted in severe learning and memory deficiency in behavioral tests. Our results also provided evidence to support the proposition that the MrD of the ventral striatum may also join the neural circuitries of the NBM in processing learning and memory signals. Since the cholinergic neurons in the basal forebrain complex are among the first group of neurons known to become degenerated in Alzheimer’s disease, the role of the new pathway between the MrD and the NBM cholinergic neurons in learning and memory may provide further insights into the pathological mechanism of Alzheimer’s disease.

## Conclusions

**Fig. 9:**
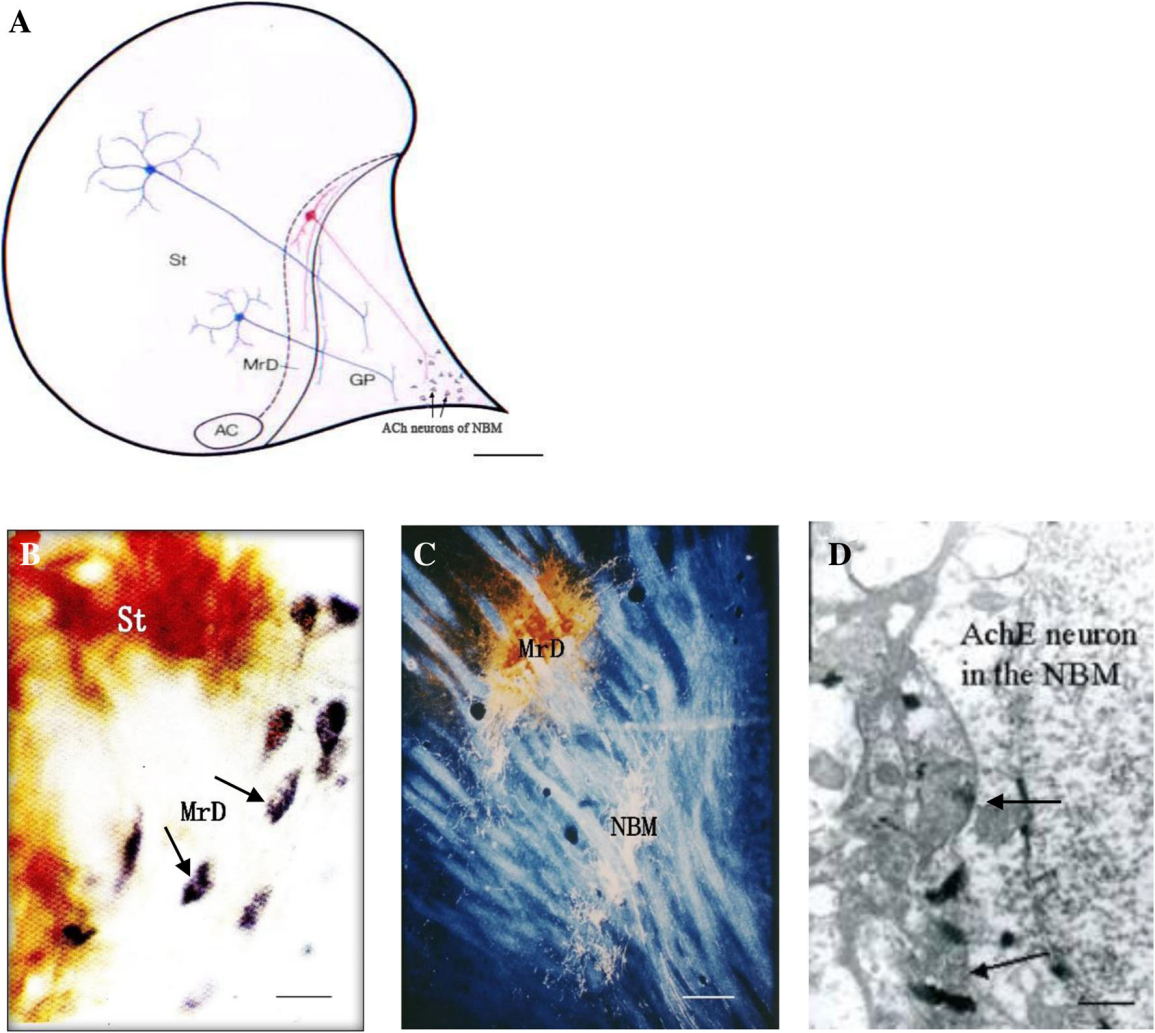
Summary of the neuronal connections from the MrD to the NBM using the WGA-HRP tracing method. Schematic summary of the connection from the fusiform neuron (red) in the marginal division (MrD) of the ventral striatum to the nucleus basalis of Meynert (NBM). The axons of round-shaped neurons (blue) in the other parts of the striatum (St) project to the globus pallidus (GP). **b** Following WGA-HRP injection to the NBM, retrogradely transported WGA-HRP, which are stained as black-blue particles, labeled some fusiform neurons in the MrD of the ventral striatum. MrD, marginal division of the striatum; NBM, nucleus basalis of Meynert. Scale bar = 100 μm. **c** The efferent projections (white lines) from the MrD to the NBM are observed in a dark field under a light microscope at low magnification. MrD, marginal division of the striatum; NBM, nucleus basalis of Meynert. Scale bar = 800 μm. **d** Following WGA-HRP injection to the MrD, anterogradely transported WGA-HRP, which appeared as rod-like electron dense substances in axon terminals from the MrD, were found in the axon-somatic synapses(arrows) of cholinergic neurons in the NBM. MrD, marginal division of the striatum; NBM, nucleus basalis of Meynert. Scare bar = 1 μm

The results of this study demonstrated a new neural pathway from the MrD of the ventral striatum to the NBM at the cellular and subcellular levels and its role in learning and memory as revealed by WGA-HRP tract tracing, histochemical staining, immunoelectronic microscopy, and Y-maze behavioral test after lesions of the MrD. Following WGA-HRP injection into the NBM, positive stained dark-blue particles of WGA-HRP were retrogradely transported to label fusiform neurons in the MrD. The WGA-HRP-labeled terminals of axons from the MrD made synaptic connections with cholinergic neurons in the NBM as demonstrated by WGA-HRP anterograde tract tracing and immunoelectronic microscopy. These results showed fusiform neurons of the MrD, some of them were cholinergic neurons, projected to the NBM and their axon terminals had synaptic connections with the cholinergic neurons of the NBM (Fig. 9). The rats with bilateral MrDs lesions showed degenerated axon terminals in synapses of the cholinergic neurons in the NBM of the rat brain under an electronic microscope and severe learning and memory deficiency of rats in the Y-maze test. The results suggested that the new neural pathway (some of them were cholinergic) from the MrD to the NBM played an important role in learning and memory. This new pathway might regulate the excitability of the NBM in the cognitive processes and probably might be involved in the complex memory networks of the brain. The new cholinergic neural pathway between the MrD and the NBM is also proposed to be potentially involved in the pathological mechanism of Alzheimer’s disease.
